# Everolimus Related Acute Steatohepatitis Detected on ^68^Ga-Dotatate PET/CT in a Patient with Metastatic Duodenal Neuroendocrine Tumor

**DOI:** 10.1007/s13139-026-00993-x

**Published:** 2026-02-27

**Authors:** John Wiltshire, Ashwin Singh Parihar

**Affiliations:** 1 Division of Nuclear Medicine, Mallinckrodt Institute of Radiology, Washington University School of Medicine, St. Louis, MO 63110 USA; 2 Siteman Cancer Center, Washington University School of Medicine, St. Louis, MO 63110 USA

**Keywords:** Everolimus, Steatosis, Hepatitis, Steatohepatitis, Neuroendocrine, Dotatate, Adverse

A 71-year-old woman with metastatic (AJCC stage IV) duodenal neuroendocrine tumor (well-differentiated, Ki-67 12%), diagnosed after extensive abdominal surgeries, was found to have liver metastases on a ^64^Cu-DOTATATE PET/CT two years after surgery (Figure 1: a-c showing the hepatic metastases with an otherwise normal hepatic parenchyma). She was initiated on everolimus (10 mg daily) with normal concurrent hepatic function tests. Follow-up ^68^Ga-DOTATATE PET/CT three months later showed stable disease (Figure 1: d-f), with marked hypoattenuation of the liver parenchyma, appearing less dense than the adjacent blood vessels (red arrow) indicating new, severe steatohepatitis. The PET images showed no visual or SUV changes in the hepatic parenchyma.

The mean liver HU on the non-contrast CT measured 5 (previously 52), compared to 43 for the spleen (previously 42). Subsequent testing showed elevated aspartate transaminase (50 units/L, previously 34) and alanine transaminase (64 units/L, previously 32) with normal alkaline phosphatase and bilirubin, compatible with grade-1 liver injury per the Drug-Induced Liver Injury Network severity grading [1]. No other significant relevant comorbidities (including diabetes, dyslipidemias, Hepatitis-B or C), significant weight gain or drug interactions were noted to explain the acute-onset severe steatohepatitis, other than the initiation of everolimus. Thus, everolimus was discontinued and octreotide was initiated. The patient translocated shortly thereafter, with no follow-up available.

Acute steatosis is a rare adverse event associated with everolimus, with a single case-report till date reporting this correlation on imaging, which was ordered because of deranged hepatic function tests and new abdominal pain [2]. However, our patient was asymptomatic and diagnosed incidentally on ^68^Ga-DOTATATE-PET/CT, prompting a change in management despite overall stable disease. Although everolimus has been associated with drug induced liver injury as an uncommon adverse effect [3], occurrence of severe steatohepatitis is rare and prior studies have hypothesized changes in insulin resistance as a possible mechanism [4]. Hepatic steatosis on a non-contrast CT appears as relative hypoattenuation of the liver compared to the spleen (liver HU atleast 10 HU below that of the spleen) and/or absolute hypoattenuation of the liver parenchyma < 40 HU. Narrower CT windowing, like that for evaluation of hepatic masses is helpful for visual analysis for detecting moderate-to-severe hepatic steatosis.

As DOTATATE-PET/CT is commonly performed for the staging and monitoring of patients with neuroendocrine tumors [5], it is important to be aware of this rare but highly relevant finding. Specifically, careful review of both the CT and PET images is crucial and can have strong therapeutic implications (Fig. 1).

**Fig. 1:**
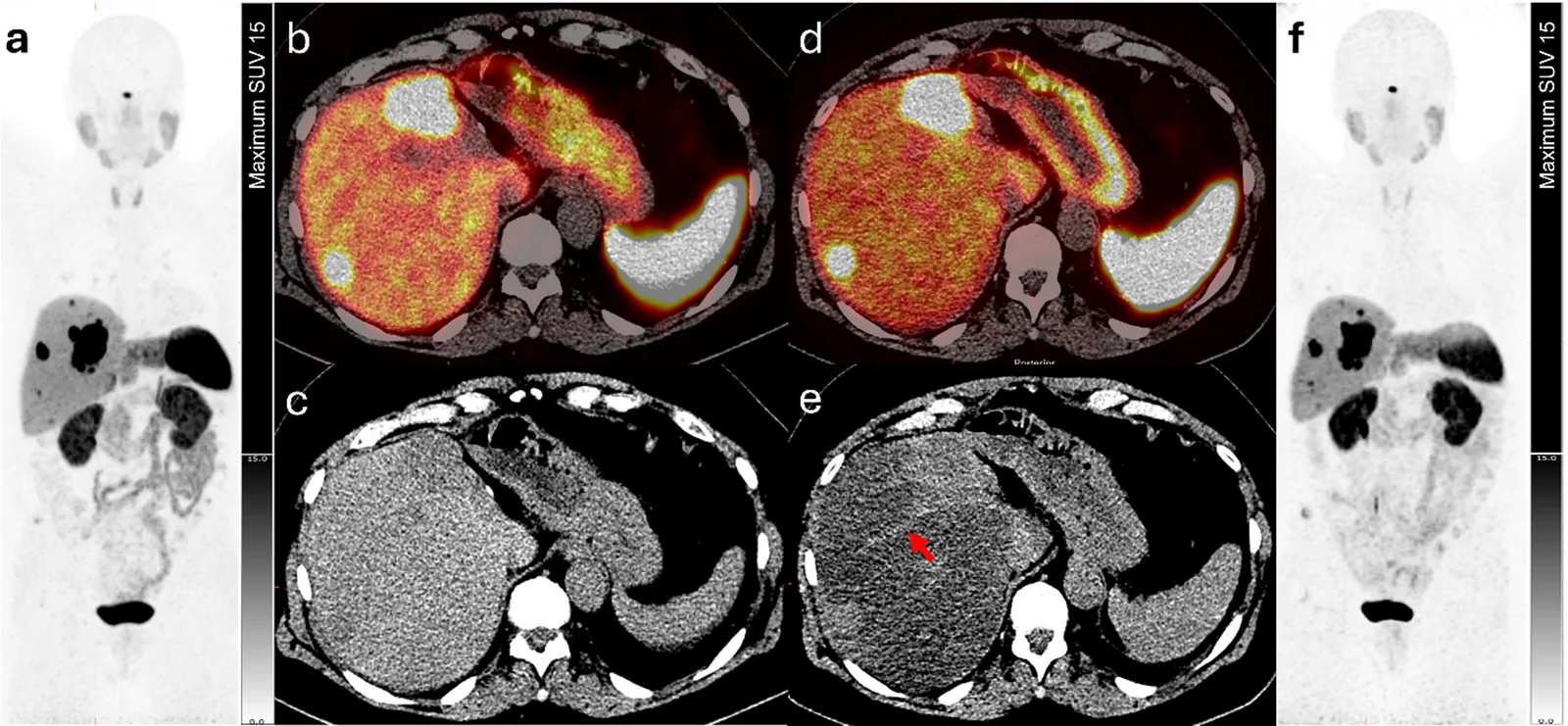
^64^Cu-DOTATATE-PET/CT (maximum intensity projection – (**a**), transaxial fused PET/CT – (**b**), CT - (**c**) showing the liver metastases from the primary neuroendocrine tumor at baseline with an otherwise normal appearing background liver parenchyma. ^68^Ga-DOTATATE-PET/CT following treatment initiation with everolimus (maximum intensity projection – (**f**), transaxial fused PET/CT – (**d**), CT - (**e**) showing marked hepatic steatosis with the hepatic parenchyma appearing less dense than the vessels (red arrow)

## Data Availability

Data sharing not applicable to this article as no datasets were generated or analyzed during the current study.
